# Comparative Effects of Fischer–Tropsch Waxes with Different Carbon-Chain Ranges on Warm-Mix Asphalt Performance: An Experimental and Molecular Dynamics Simulation Study

**DOI:** 10.3390/ma19163372

**Published:** 2026-08-07

**Authors:** Chengqin Chen, Wei Zhang, Chenggui Chen, Hongjuan Wu, Rui Wang, Xiaoyan Ma, Xiaolei Wu

**Affiliations:** 1 School of Civil Engineering, Northwest Minzu University, Lanzhou 730030, China; whj0116@163.com (H.W.); 13659464950@163.com (R.W.); 2 Gansu Provincial Transportation Research Institute Group Co., Ltd., Lanzhou 730030, China; 17862980373@163.com; 3 School of Materials Science and Engineering, Chang’an University, Xi’an 710064, China; xiaoyanma@chd.edu.cn (X.M.); 2025231076@chd.edu.cn (X.W.)

**Keywords:** SBS-modified asphalt, viscosity–temperature relationship, rheological properties, molecular dynamics simulation, cohesive energy density, free-volume fraction

## Abstract

Fischer–Tropsch (FT) wax is widely used as an organic warm-mix asphalt (WMA) additive, lowering binder viscosity during construction while improving high-temperature deformation resistance in service; however, the comparative responses of SBS-modified asphalt to different FT wax grades remain insufficiently understood. Sasobit and three FT waxes with different carbon-chain ranges (FT 80, FT 90, FT 100) were incorporated into SBS-modified asphalt at about 7.0 wt%, and their effects on macroscopic performance, rheology, molecular packing, and diffusion were evaluated using physical-property tests, rotational viscosity, dynamic shear rheometer (DSR) testing, and molecular dynamics (MD) simulation. In the MD analysis, the wax additives were represented by linear alkane molecules with different chain lengths, and the systems were subjected to structural optimization, annealing, and NPT equilibration using the COMPASS III force field before the molecular descriptors were evaluated. The experimental results showed that all four additives produced a trade-off between increased high-temperature stiffness and reduced low-temperature ductility. Sasobit gave the strongest viscosity reduction (>70% above 165 °C), while FT 90 and FT 100 showed more stable, predictable viscosity–temperature behavior favorable for a wider construction window. DSR results showed higher complex modulus and lower phase angle for all modified binders at low frequencies, suggesting an increased elastic contribution and greater resistance to deformation under the tested rheological conditions; FT 80 produced the greatest stiffening but also the largest free volume and loosest molecular packing, whereas FT 100 increased cohesive energy density and reduced free volume, reflecting denser packing and stronger intermolecular cohesion. MD simulations revealed that FT wax enhanced short-time local molecular mobility and segment diffusion in its molten state (explaining the warm-mix viscosity reduction), whereas macroscopic stiffening and ductility loss at ambient temperatures were dictated by wax microcrystallization and physical network constraints that restricted long-range chain relaxation. By comparing three FT wax grades and Sasobit under the same experimental dosage and testing framework, this study provides a controlled assessment of the relationships among wax-grade characteristics, binder-scale rheological responses, and MD-derived molecular descriptors.

## 1. Introduction

Asphalt concrete is the most widely used material for road surface construction globally, valued for its durability, noise-dampening properties, and adaptability to diverse traffic and climatic conditions. Conventional hot-mix asphalt (HMA) production and placement, however, require aggregate mixing temperatures of 160~190 °C, leading to substantial fuel consumption, carbon dioxide emissions, and the generation of volatile organic compounds (VOCs) and asphalt fumes hazardous to construction workers [1,2]. In the context of growing regulatory pressure to decarbonize construction activities and international commitments to reduce greenhouse gas emissions, the asphalt industry has pursued strategies to lower production temperatures without sacrificing mechanical performance.

Warm-mix asphalt (WMA) technology addresses this challenge by enabling mixing, placement, and compaction at temperatures 20~40 °C below those of conventional HMA, achieved through the use of chemical or physical additives that temporarily reduce binder viscosity during construction [3]. Among the available WMA technologies, Fischer–Tropsch (FT) wax has attracted sustained research interest owing to its temperature-dependent effects: above its melting or dissolution range, it can reduce binder viscosity, whereas crystallization during cooling can increase binder stiffness [4,5]. Commercial FT waxes traditionally utilized in asphalt modification (such as Sasobit) consist of long-chain linear alkanes predominantly spanning C_45_ to C_115_. Produced via the Fischer–Tropsch synthesis process, the catalytic conversion of syngas (CO + H_2_), these synthetic waxes exhibit high structural regularity and compositional tunability, which are unattainable with petroleum-derived paraffin waxes [6].

Extensive laboratory testing has demonstrated that Sasobit addition consistently stiffens the asphalt binder: penetration at 25 °C decreases by 15~45% and ring-and-ball softening point increases by 5~15 °C relative to the base binder, with both effects intensifying at higher wax dosages [4,5]. However, this stiffening effect may be accompanied by reduced low-temperature ductility, raising concerns about thermal cracking susceptibility in cold climates [7,8,9,10,11]. The magnitude of ductility loss is sensitive to both wax content and base asphalt grade, making it a critical constraint in the dosage optimization of FT wax modifiers.

The warm-mix functionality of FT wax derives from a pronounced and temperature-sensitive reduction in binder viscosity above the wax melting point. Akisetty et al. [12] measured rotational viscosity for Sasobit-modified binders and reported 25~50% reductions at 120~160 °C relative to the unmodified base, enabling a mixing and compaction temperature reduction of approximately 20~30 °C without sacrificing aggregate coating quality or compaction density. At service temperatures, Sasobit addition may increase zero-shear viscosity and binder stiffness after wax crystallization [13]. Goh et al. [14] compared multiple organic wax additives and confirmed that FT wax achieves greater absolute viscosity reduction above the melting point than amide-based waxes, attributing this advantage to the higher n-paraffin purity and sharper melting transition of FT wax.

Dynamic shear rheometer (DSR) testing is widely used to characterize the linear viscoelastic response of unmodified and modified asphalt binders [15,16]. Sasobit addition consistently increases the complex modulus G* and decreases the phase angle δ at high temperatures, elevating the rutting factor G*/sinδ by one to two PG grades [5,7,9,17]. Frequency sweep and master curve analyses have been used to evaluate the rheological response of wax-modified binders over extended loading-time ranges [18,19]. Multiple stress creep and recovery (MSCR) testing, increasingly preferred over the traditional G*/sinδ criterion as a predictor of field rutting [20], has demonstrated that FT wax substantially reduces non-recoverable creep compliance (Jnr) and increases percent recovery (%R), indicating greater elastic recovery under the tested MSCR conditions [21]. However, wax-induced stiffening does not translate uniformly into fatigue performance at intermediate temperatures. The Linear Amplitude Sweep (LAS) test provides a damage-based evaluation by applying progressively increasing strain amplitudes and estimating fatigue life within a viscoelastic continuum damage framework. LAS-based studies have shown that the fatigue response of wax-modified binders depends on wax molecular structure, binder composition, aging condition, and loading level: triacontane reduced the fatigue resistance of model asphalt, whereas other molecular configurations produced different responses [22], and Sasobit improved fatigue life in some polymer-modified binders, although its effect varied with polymer type and aging condition [23]. FT wax dosage has also exhibited strain-dependent effects, indicating that fatigue behavior cannot be inferred solely from conventional linear viscoelastic parameters [24]. The compatibility of FT wax with asphalt during high-temperature storage is governed by the colloidal structure of the binder. In practice, Sasobit-modified binders have shown softening point differentials (ΔSP) below 2 °C after 48 h storage at 163 °C, satisfying the EN 13399 criterion and indicating adequate short-term compatibility [25,26]. However, Biro et al. [7] cautioned that stability deteriorates at wax dosages above 4% and in softer base asphalts, underscoring the sensitivity of storage stability to both wax content and base asphalt composition.

The emergence of molecular dynamics (MD) simulation has opened a pathway to mechanistic understanding of wax–asphalt interactions at the nanoscale. Li and Greenfield [27] developed a validated 12-component asphalt molecular model, benchmarked against experimental density, solubility parameter, and diffusion coefficients, that has become the standard reference framework for asphalt modifier simulation. Building on this foundation, Pan and Tarefder [28] demonstrated that van der Waals forces dominate wax asphalt interactions, with n-alkane molecules preferentially associating with saturate and aromatic components over polar asphaltenes. Zhang et al. [29] showed that longer-chain wax molecules form more cohesive crystalline aggregates in the asphalt matrix, producing stronger crystal networks and greater high-temperature stiffening. Xu et al. [30] used radial distribution functions and mean squared displacement analysis to characterize modifier diffusivity and spatial organization within the asphalt system, while Yao et al. [31] provided a comprehensive methodological review affirming MD simulation as a reliable and increasingly powerful tool for asphalt modifier research when force fields are appropriately selected and simulation parameters rigorously validated. More recently, Xing et al. [32] reviewed the broader application of MD simulation to asphalt materials, covering high-temperature stability, low-temperature flexibility, self-healing behavior, aging, and interfacial adhesion, and confirmed that MD-derived molecular indicators are increasingly used to complement macroscopic performance testing across the asphalt modifier literature. Nevertheless, simplified molecular models do not reproduce the complete compositional distributions of commercial wax products [22].

Despite this body of work, the effects of representative FT wax chain length under consistent binder, dosage, preparation, and testing conditions remain insufficiently understood [9,33]. First, virtually all published studies treat FT wax as a single additive class represented by Sasobit or a small number of commercial grades with similar chemical profiles. Direct comparisons among multiple FT wax grades under the same binder composition, additive dosage, preparation procedure, and testing conditions remain limited. Controlled comparisons are therefore needed to distinguish wax-grade-related responses from differences caused by binder source, dosage, preparation, or testing protocol. Second, the integration of macroscopic performance characterization with molecular-scale simulation for structurally diverse FT waxes remains undeveloped. Existing MD studies have examined selected wax–asphalt systems, but direct comparisons among multiple FT grades within a consistent experimental and modeling framework remain limited [22,29]. This limits the interpretation of relationships between MD-derived descriptors and experimentally measured binder responses.

To address these gaps, the present study compares three Fischer–Tropsch wax grades (FT 80, FT 90, and FT 100) with Sasobit under the same additive dosage, binder composition, preparation procedure, and testing framework. At the macroscopic level, penetration, softening point, and ductility tests evaluate modifications to conventional physical properties; rotational viscosity measurements across a broad temperature range establish viscosity–temperature relationships; and DSR testing characterizes complex modulus and phase angle of the control and wax-modified binders. At the molecular level, MD simulation is employed to evaluate cohesive energy density, fractional free volume, mean squared displacement, and diffusion-related descriptors in the different wax-modified asphalt systems. By comparing molecular-scale descriptors with macroscopic performance metrics across the investigated wax additives, this study examines the relationships between molecular characteristics and binder-scale responses. The findings are intended to provide additional insight into FT wax–asphalt interactions and a comparative basis for evaluating the investigated additives under the fixed dosage and test conditions.

## 2. Materials and Methods

### 2.1. SBS Modified Asphalt and WMA Additives

SBS-modified asphalt was selected as the base asphalt in this study to investigate the effects of the Sasobit warm-mix additive and Fischer–Tropsch wax on its properties. SBS-modified asphalt was chosen as the matrix binder because it remains the most widely used polymer-modified binder in high-performance pavement construction, and its warm-mix behavior when combined with organic wax additives has recently attracted renewed research attention [34]. The SBS-modified asphalt was prepared by blending No. 90 base asphalt with 5% SBS modifier, followed by shearing, swelling, and maturation. The properties of the base asphalt and the corresponding SBS-modified asphalt are presented in Table 1.

### 2.2. FT Waxes and Sasobit Additive

The Sasobit warm-mix additive (Sasol Wax, Sasolburg, South Africa) and the FT 80, FT 90, and FT 100 products (Shengzhou Worthside Chemical Co., Ltd., Shaoxing, China) investigated in this study are Fischer–Tropsch wax additives composed predominantly of long-chain saturated hydrocarbons. Their principal hydrocarbon components follow the general molecular formula *C_n_H*_2*n*+2_, although commercial products may contain distributions of chain lengths and minor constituents. Their carbon skeletons adopt a neat, zigzag planar conformation during solid-state crystallization. Owing to their long-chain hydrocarbon composition, they exhibit relatively low viscosity in a molten state. The hydrocarbon chains are held together primarily by van der Waals forces generated when the long chain segments align parallel to one another. The strength of these interactions generally increases with molecular chain length.

The most fundamental difference among them lies in the distribution range of their molecular chain lengths and the macroscopic melting points determined by it. FT 80, FT 90, and FT 100 are general-purpose industrial base waxes whose carbon numbers and melting points increase progressively with their grade numbers. Their carbon numbers are primarily concentrated in the ranges of C_30_~C_60_, C_40_~C_80_, and C_45_~C_100_, respectively, with corresponding melting points ranging roughly from 80 °C to 100 °C. In contrast, Sasobit, a specialized warm-mix additive for asphalt, features a specially screened “broad-narrow composite” ultra-long chain structure. It possesses a broader carbon number distribution (mainly between C_40_~C_115_) and contains a significantly higher proportion of ultra-long linear molecules above C_100_. This unique molecular weight distribution allows Sasobit to not only provide excellent viscosity-reducing lubrication for asphalt at high temperatures above 115 °C through its specific ratio of long and short chains, but also form a tougher micro-crystalline grid via its ultra-long carbon chains at temperatures below 100 °C, thereby preventing asphalt pavements from softening and rutting under high summer temperatures. The physicochemical Properties of FT Waxes and Sasobit Additive are listed in Table 2.

### 2.3. Construction of Complex Modulus and Phase Angle Master Curves

To determine the linear viscoelastic (LVE) range of the aged asphalt, strain-controlled amplitude sweep tests were conducted prior to the frequency sweep measurements. During the amplitude sweep test, the loading strain was gradually increased while maintaining a constant loading frequency and test temperature. The complex shear modulus (G*), and phase angle (δ), were continuously monitored to evaluate the response of the material under increasing deformation.

At relatively low strain levels, the asphalt exhibited a stable viscoelastic response, characterized by nearly constant G* and δ values. This range was defined as the LVE region, in which the material response is independent of the applied strain amplitude and no significant damage or microstructural disturbance occurs. As the strain level increased, a noticeable reduction in G* indicated the onset of nonlinear behavior and structural damage within the asphalt.

In this study, the upper limit of the LVE range was identified as the strain level corresponding to a 5% reduction in the initial complex shear modulus. The strain amplitude selected for the subsequent frequency sweep tests was required to remain below this limit to ensure that all rheological measurements were conducted within the LVE region.

Based on the test results obtained at a loading frequency of 1 Hz, the LVE strain limits of the different asphalt at temperatures ranging from 20 °C to 80 °C varied from 1% to 22%. To ensure that all frequency sweep measurements for all specimens and test temperatures were performed within the LVE region, a strain amplitude of 1% was selected for the subsequent frequency sweep tests. The frequency sweep tests were conducted from 20 °C to 80 °C at 10 °C intervals, over an angular frequency range of 0.1 to 100 rad/s.

The G* and δ obtained from the frequency sweep tests at different temperatures were used to construct rheological master curves based on the time–temperature superposition principle; the same frequency sweep and master curve procedure has been applied to warm-mix and Sasobit-modified binders in recent rheological studies [35]. A reference temperature of 40 °C was selected. For each test temperature, the corresponding frequency sweep data were horizontally shifted along the logarithmic frequency axis to align with the response measured at the reference temperature. The horizontal shift factors were determined using the least-squares method. Specifically, the shift factor at each temperature was adjusted to minimize the sum of squared differences between the shifted experimental data and the reference-temperature data within their overlapping frequency range (Equation (1)).

(1) $$\omega_{r}=\alpha_{T}\omega$$

where $\omega_{r}$ is the reduced angular frequency, ω is the measured angular frequency, and $\alpha_{T}$ is the horizontal shift factor at temperature T. At the reference temperature of 40 °C, the shift factor was defined as ($\alpha_{T}$ = 1).

After applying the optimized shift factors, the G* and δ obtained at all test temperatures were combined to generate the master curves of G* and δ, respectively. These master curves describe the viscoelastic response of the asphalt over an extended frequency range and enable the rheological behavior of different materials to be compared under equivalent loading-time conditions. All experimental measurements were performed in triplicate, and the results are reported as the mean.

### 2.4. Molecular Model Construction of SBS/WMA Composite Modified Asphalt

**Figure 1:**
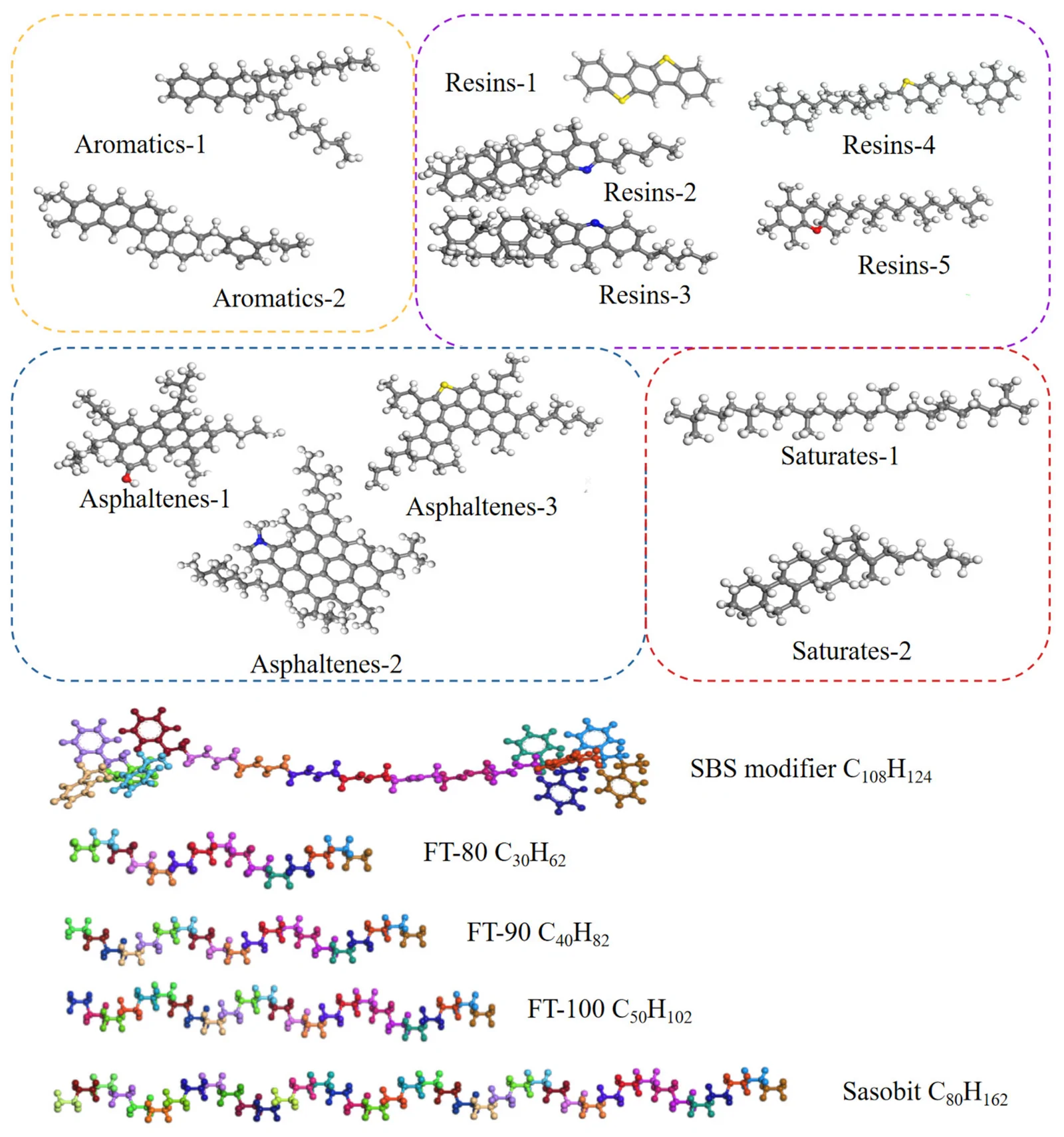
Molecular structure models of asphalt SARA fractions and modifiers used in molecular dynamics simulations.

To simulate the molecular dynamics of warm-mix asphalt, following the modeling approach previously used to investigate the adhesion performance of wax-based warm-mix asphalt at the molecular scale [36], a multi-component AAA (SARA) framework was established to represent the matrix asphalt, as illustrated in the top portion of Figure 1. The base asphalt model is comprehensively composed of four distinctive chemical fractions: three polycyclic aromatic molecules containing heteroatoms (Asphaltenes-1 to Asphaltenes-3) representing the highly polar asphaltene core; five diverse molecular structures (Resins-1 to Resins-5) with intermediate polarity and various heteroatoms acting as the resin compatibilizer phase; two condensed aromatic structures with aliphatic side chains (Aromatics-1 and Aromatics-2) serving as the aromatic dispersing medium; and a long straight-chain alkane along with a fused ring system (Saturates-1 and Saturates-2) to accurately constitute the saturates fraction.

As shown in Figure 1, four straight-chain saturated alkane molecules with systematically varied chain lengths were introduced into the asphalt matrix as simplified representative models of the investigated wax additives. The three primary industrial-grade Fischer-Tropsch waxes, FT 80, FT 90, and FT 100, are explicitly modeled by triacontane (C_30_H_62_), tetracontane (C_40_H_82_), and pentacontane (C_50_H_102_), respectively, capturing the progressive increase in their molecular weight and carbon number. In contrast, the commercial benchmark Sasobit warm-mix additive is represented by a significantly longer octacontane (C_80_H_162_) chain, enabling a comparative molecular dynamics analysis regarding how different linear chain lengths and their respective van der Waals crystallization networks influence the high-temperature viscosity reduction and anti-rutting behavior of the modified binder. These simplified single-molecule models were used to compare representative chain-length effects and do not reproduce the complete carbon-number distributions, polydispersity, branching, or minor constituents of the corresponding commercial wax products.

In this study, The dosage of the four organic wax WMA additives (FT80, FT90, FT100, and Sasobit) was designed targeting a representative high-concentration regime (averaging~7.0%) by total weight of the modified binder system). This specific concentration range (6.13% to 9.42%) was selected because it reflects the upper operational limit frequently evaluated for organic wax modifiers in practical pavement engineering; it maximizes high-temperature viscosity-reduction efficiency and rutting resistance while magnifying the underlying molecular mechanisms for comparative analysis. In MD simulation systems, discrete molecule counts (6 to 10 additive molecules) were incorporated into the SBS-modified asphalt matrix. Maintaining a sufficiently high molecule count across all models prevents localized spatial variance and statistical fluctuations within the periodic boundary conditions. Due to the significant variation in molecular weights among the waxes (ranging from C_30_H_62_ at 422.81 g/mol to C80H162 at 1122.37 g/mol), the precise mass fractions are 6.13% (FT80), 7.26% (FT90), 7.06% (FT100), and 9.42% (Sasobit), as detailed in Table 3. By anchoring all systems within this characteristic high-dosage regime, the influence of systematic variations in linear chain lengths (C_30_, C_40_, C_50_, and C_80_) and their respective van der Waals networks on the thermodynamic and rheological properties of the WMA binders can be evaluated directly and transparently.

**Figure 2:**
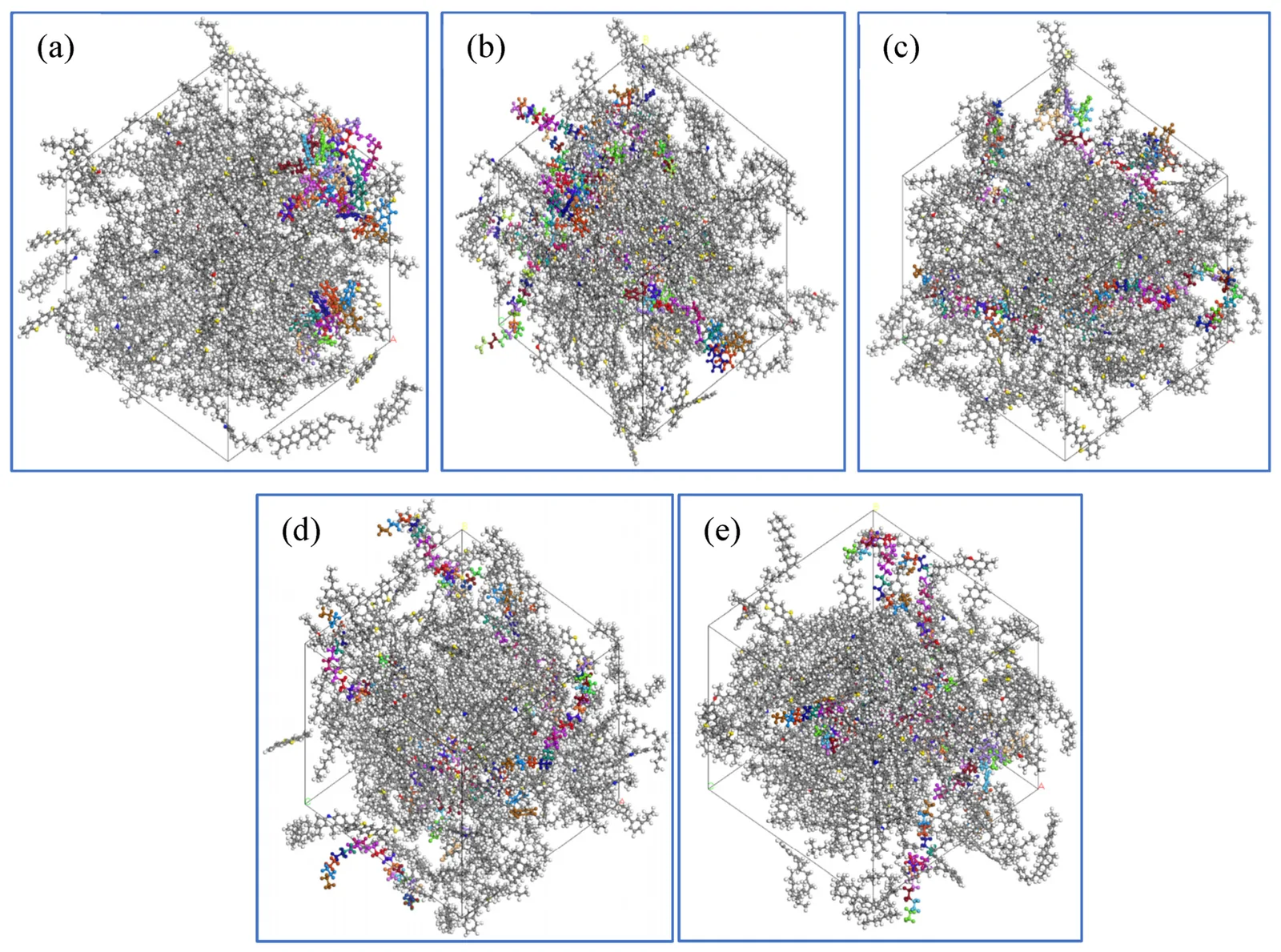
Molecular dynamics simulation cell of the asphalt–modifier composite system; (**a**) SBS asphalt; (**b**) Sasobit; (**c**) FT 100; (**d**) FT 90; (**e**) FT 80.

A styrene-butadiene-styrene (SBS) triblock copolymer modifier was incorporated into the SARA matrix to construct an SBS-modified asphalt model. As shown in Figure 1, the SBS modifier is represented by a representative linear chain structure with a chemical formula of C_108_H_124_. This molecular model captures the classic block features of the thermoplastic elastomer, consisting of rigid polystyrene (PS) end blocks that provide mechanical strength and a flexible polybutadiene (PB) mid-block that imparts elasticity. In the simulation, the SBS molecules form an interwoven elastomeric network within the 12-component asphalt matrix. The molecular structure models of asphalt SARA fractions and modifiers used in molecular dynamics simulations are shown as Figure 1. Following the establishment of the SBS-modified asphalt matrix, the four organic wax WMA additives (FT 80, FT 90, FT 100, and Sasobit) were subsequently introduced at a fixed dosage of 3.0 wt% by weight of the entire modified binder system. The molecular components of SBS asphalt and wax-modified asphalt models and the molecular dynamics simulation cell of the asphalt–modifier composite system are shown as Table 3 and Figure 2. This composite modeling approach allows for a comprehensive molecular dynamics evaluation of the interactive mechanisms between the linear wax crystal growth and the polymer-asphalt network.

Molecular dynamics (MD) simulations were performed via BIOVIA Materials Studio (Dassault Systèmes BIOVIA, San Diego, CA, USA; https://www.3ds.com/products-services/biovia/products/molecular-modeling-simulation/, accessed on 3 August 2026) with the COMPASS III force field at medium-accuracy settings. The system was first subjected to geometry optimization through a stepwise optimization protocol, beginning with the Steepest Descent algorithm, followed by the Adjusted Basis Newton-Raphson (ABNR) method, and finally refined using the Quasi-Newton approach. Subsequently, simulated annealing was applied to overcome local energy traps and improve the molecular packing configuration. The annealing procedure was performed over a temperature interval of 300~600 °C and consisted of five cycles, each including an MD run followed by structural optimization. In each cycle, the MD simulation was carried out for 200 ps, while the following geometry optimization was limited to 10,000 iterations. After annealing, dynamic equilibration was performed in the isothermal-isobaric (NPT) ensemble with a time step of 1 fs for 1000 ps. The temperature and pressure were controlled by the Nosé–Hoover thermostat and Berendsen barostat, respectively. For non-bonded interactions, van der Waals interactions were treated using the particle–particle particle–mesh (PPPM) method, whereas electrostatic interactions were calculated with an atom-based summation approach.

## 3. Results and Discussion

### 3.1. Conventional Performance Characterization

**Figure 3:**
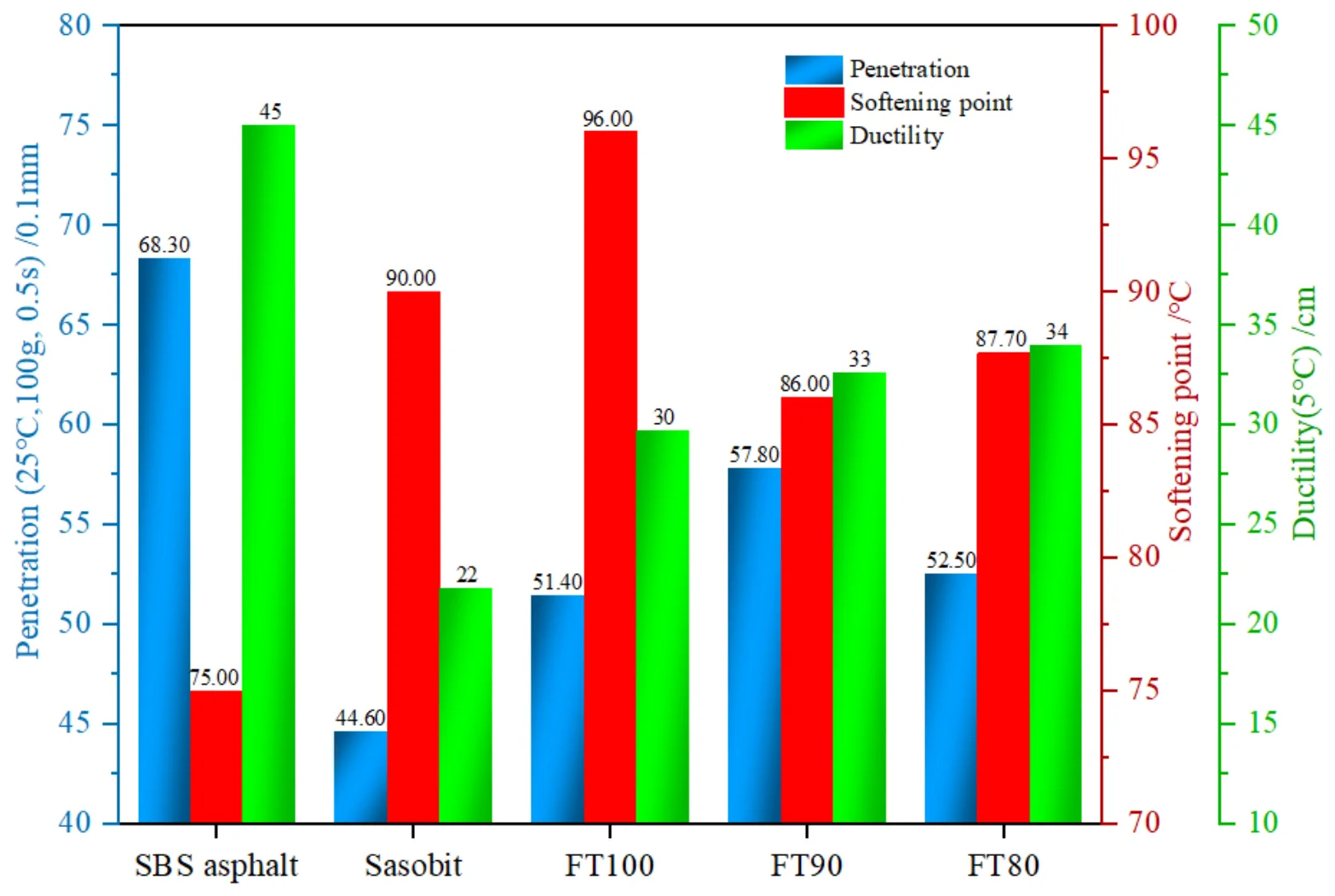
Penetration, Softening Point, and Ductility of Different Modified Asphalts.

The conventional physical-property results are presented in Figure 3. All ductility values shown in Figure 3 were measured at 5 °C under the same test conditions. The results show that the incorporation of wax-based additives reduced the low-temperature ductility of the SBS-modified binder. Experimental data indicated a precipitous decline in ductility at 5 °C across all modified groups, dropping from 45 cm for the control SBS-modified bitumen to a range of 21.8 cm to 34 cm. Concurrently, a substantial increase in stiffness was observed; the penetration values decreased across all modified groups, with the Sasobit group showing the most pronounced reduction of 34.7%, while softening points escalated significantly, with both the Sasobit and FT 100 groups surpassing 90 °C. These results demonstrate that while these additives effectively enhance high-temperature stiffness and rutting resistance, they impose a severe penalty on the material’s stress-relaxation capacity at low temperatures, thereby exacerbating the risk of brittle fracture. A recent study of AC30 binders with and without 40% RAP binder similarly showed that the addition of 2% FT wax reduced viscosity and improved high-temperature rutting resistance but deteriorated intermediate-temperature fracture resistance, while the relative low-temperature performance varied with wax type and RAP condition [11]. Comparable penetration and softening-point trends have also been reported for high-viscosity polymer asphalt binders modified with Fischer-Tropsch wax of different melting points, where the negative effect on low-temperature performance was found to outweigh the high-temperature benefit at higher wax dosages [10].

The observed performance evolution is primarily attributed to the unique “crystallization-melting” behavior of the wax fractions within the bitumen matrix. During the hot-mix stage, the wax components melt rapidly, forming a low-viscosity fluid that promotes homogeneity and improves workability. However, as the service temperature decreases, these wax molecules undergo a phase transition, precipitating to form a structured microcrystalline network. These crystalline domains function as physical cross-linking points within the bitumen matrix, exerting a “pinning effect” on the bitumen molecular chains and the SBS polymer network, which restricts their free movement and significantly elevates macro-scale stiffness. This microcrystalline lattice not only occupies the free volume of the matrix but also disrupts the ductile deformation pathways of the bitumen under tensile stress. Consequently, the material loses its ability to dissipate energy through molecular rearrangement at low temperatures, leading to the observed impairment in ductility and a reduced fracture toughness.

### 3.2. Viscosity–Temperature Susceptibility

**Figure 4:**
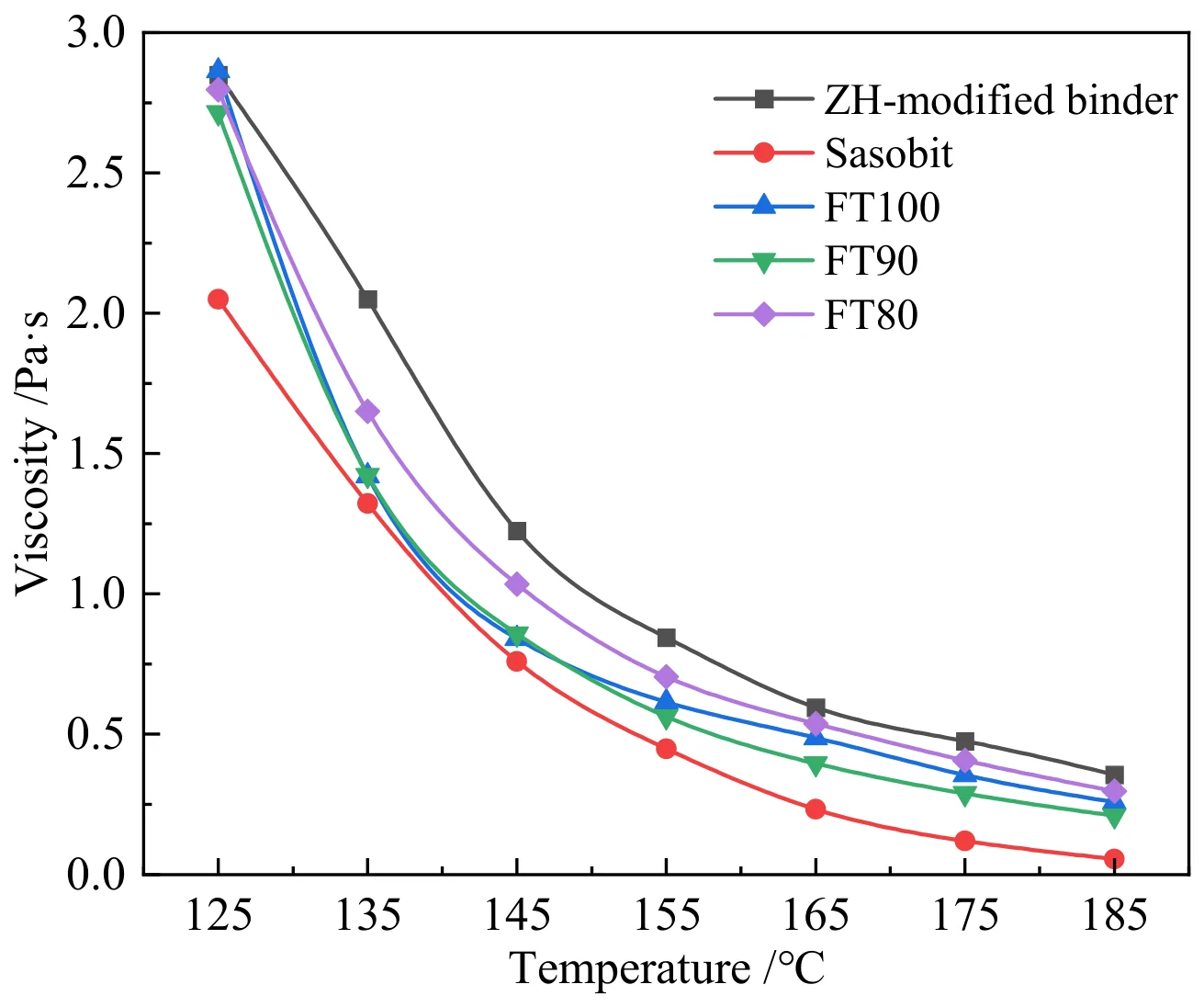
Viscosity–Temperature Curves of Different Modified Asphalts.

The rotational-viscosity measurements, conducted across a temperature range of 125 °C to 185 °C using a Brookfield viscometer (Brookfield Engineering Laboratories, Middleboro, MA, USA) equipped with an SC4-18 spindle at a rotational speed of 10 rpm, demonstrate that the incorporation of Sasobit and various FT waxes significantly reduces the construction viscosity of the base SBS-modified bitumen as Figure 4. Sasobit exhibits the most profound viscosity reduction efficiency, particularly at elevated temperatures exceeding 165 °C, where it achieves a reduction of over 70% compared to the control binder. This substantial attenuation of flow resistance provides a marked improvement in material workability, directly facilitating lower energy consumption during mixing and paving operations and validating the efficacy of these additives in modern warm-mix asphalt applications. A similarly pronounced viscosity reduction above the wax melting point has been reported for other wax-based warm-mix additives in high-viscosity modified asphalt, together with corresponding changes in aging susceptibility, confirming that the magnitude of viscosity reduction is closely tied to the melting behavior of the specific wax grade rather than being a generic wax effect [37].

Further quantitative assessment using the Viscosity–Temperature Susceptibility (VTS) index confirms the functional superiority of these additives in stabilizing rheological performance. The FT wax series, specifically FT 90 and FT 100, demonstrates a stable and linear viscosity reduction trend within the 145 °C~175 °C interval, establishing a theoretical basis for energy-efficient pavement construction. By narrowing the viscosity variance across varied thermal profiles, these agents effectively broaden the construction window, thereby enhancing temperature stability during the compaction phase and ensuring the uniformity of the mechanical properties within the resulting asphalt layer.

A comparative analysis of the viscosity–temperature profiles reveals distinct modification mechanisms between Sasobit and the FT wax series. Sasobit exhibits non-linear rheological attenuation, characterized by a rapid viscosity drop upon reaching its melting point; this thermal trigger effect is advantageous for immediate workability at critical compaction temperatures, albeit at the cost of a sharper viscosity gradient that demands stringent thermal control. Conversely, FT 90 and FT 100 demonstrate superior linear stability in their viscosity–temperature curves, signifying a more predictable rheological response. Compared to Sasobit, the FT waxes exhibit a more tempered reduction in VTS, providing a balanced compromise between lowering construction energy requirements and maintaining the binder’s structural stability against ambient thermal fluctuations.

### 3.3. Complex Modulus Master Curves and Hardening Mechanism

The complex modulus (G*) master curves were constructed using the time–temperature superposition principle (TTSP) based on dynamic shear rheometer (DSR, Anton Paar GmbH, Graz, Austria) testing and horizontal shifting, comprehensively reflecting the stiffness evolution of the five asphalt binders over a wide range of frequencies and temperatures. A reference temperature of 40 °C was selected, and the corresponding logarithmic shift factors at different temperatures are presented in Table 4. The master curves indicate that, across the entire frequency spectrum, the modified asphalts incorporating Sasobit and the three types of FT waxes (FT 80, FT 90, and FT 100) exhibit higher complex moduli than the pristine SBS-modified asphalt without any warm-mix additives. This stiffness enhancement is particularly pronounced in the low-frequency region, corresponding to high temperatures or low loading rates. Conversely, as the frequency extends toward the high-frequency region, corresponding to low temperatures or high loading rates, the master curves of all five materials gradually converge toward the glassy modulus. This convergence demonstrates that the adverse hardening effect of the warm-mix additives on the low-temperature stiffness of the asphalt is relatively limited and that their primary modification effect is concentrated in the intermediate- to high-temperature range.

**Figure 5:**
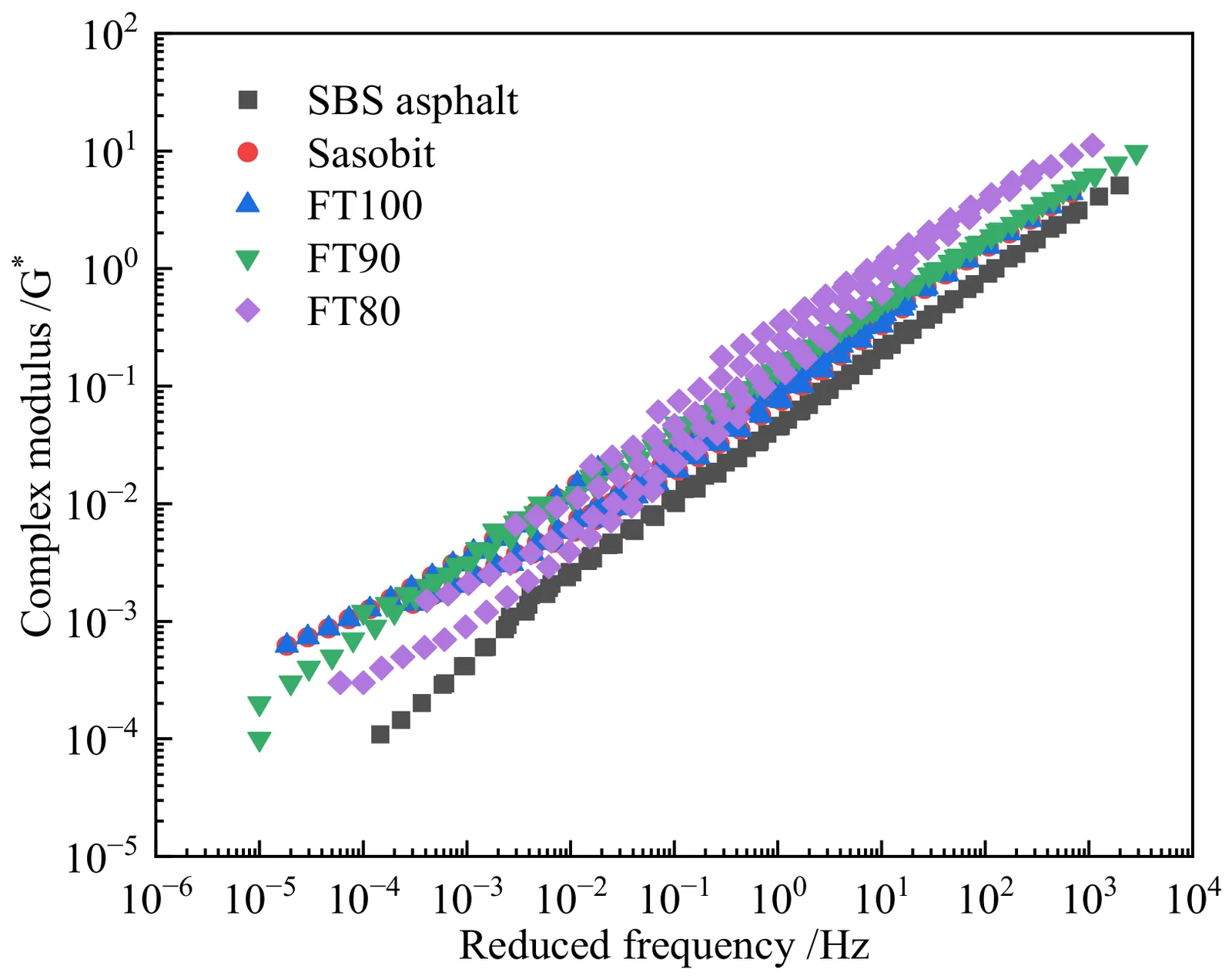
Complex Modulus Master Curves of Different Modified Asphalts.

In the low-frequency and high-temperature region, the remarkable increase in the complex modulus of the modified asphalts unveils the unique “hardening mechanism” of the small-molecule crystalline additives. In this regime, the base SBS-modified asphalt matrix undergoes severe stiffness attenuation due to the intensified viscous flow of light components, dropping its complex modulus to the order of 10^−4^ MPa. However, the four binders blended with Sasobit and FT waxes successfully maintain their low-frequency moduli above 10^−3^ MPa, representing a several-fold to ten-fold increase compared to the control SBS-modified asphalt (Figure 5). The micro-rheological essence of this behavior lies in the fact that Sasobit and FT waxes are long-chain aliphatic crystalline hard waxes with melting points typically around 100 °C. At the operating temperatures corresponding to the master curves, these additives completely precipitate from the asphalt matrix as solid microcrystals, interlocking to construct a microscopic crystalline lattice network. This rigid crystalline skeleton effectively locks the light oils within the asphalt, restrains the relative slippage among viscous fluid molecules, and intertwines with the existing swollen SBS polymer network. Consequently, a stronger composite spatial load-bearing structure is formed, imparting excellent high-temperature rutting resistance to the materials. A similar crystalline-network stiffening mechanism has been reported for warm-mixed, fast-melting SBS-modified asphalt, where atomic force microscopy and DSR testing jointly confirmed that the precipitated wax phase reinforces the swollen SBS network rather than substituting for it [34].

A longitudinal comparison of the master curves across the entire frequency range reveals that the contribution of different additives to the stiffness of the modified asphalt follows the sequence of: FT 80 > FT 90 > FT 100 ≈ Sasobit > SBS Modified Asphalt. This variance is governed primarily by the molecular weight distribution, carbon chain length, and corresponding crystallinity of the different warm-mix additive grades. Owing to its specific carbon chain configuration, FT 80 forms a more rigid and fully precipitated lattice network within the testing environment, thereby manifesting the most potent hardening effect and the highest modulus values. As the wax grade number increases (shifting from FT 80 to FT 100), the proportion of high-melting-point long-chain aliphatic hydrocarbons in the composition is finely tuned. This modification sequentially diminishes the rigidity of the microscopic crystalline network formed in the asphalt matrix, causing its master curve trajectory to gradually approach those of the commercial warm-mix agent Sasobit and the base SBS asphalt binder.

### 3.4. Characteristics of Phase Angle Master Curves and Viscoelastic Transition Mechanism

As a core rheological indicator characterizing the viscoelastic ratio of asphalt materials, the phase angle (δ) master curve intuitively reflects the internal structural response under varying temperatures and shear rates. The master curve results show prominent phase-specific discrepancies in the δ trajectories of the five asphalt binders across the frequency domain. In the high-frequency zone (corresponding to low temperatures or high-frequency loads), all materials converge to a phase angle of approximately 40°~50°. This convergence implies that when approaching the glassy transition zone, the viscoelastic features of the base asphalt matrix tend to be identical, and the influence of the additives ceases to be dominant. However, as the frequency decreases toward the intermediate-to-low frequency region (corresponding to intermediate-to-high temperature environments), the master curves of the SBS modified asphalt and those with warm-mix additives undergo a drastic divergence, displaying entirely distinct rheological evolution characteristics.

In the intermediate-to-low frequency region, the sharp variation in the phase angle unveils a competitive and synergistic mechanism in the viscoelastic transition between the high-molecular-weight modifiers and the small-molecule crystalline additives. At the low-frequency end, the SBS-modified asphalt exhibits a broad elastic plateau (maintaining between 55° and 66°) as the temperature rises, which indicates that the swollen SBS polymer network provides a delayed elastic rebound when the asphalt matrix thins out. Conversely, with the introduction of Sasobit and FT 90 and FT 100, the phase angle at the ultra-low frequency end is strongly suppressed and restricted to an extremely low level of 33°~40°, which is significantly lower than that of the SBS modified asphalt. The underlying micro-rheological mechanism is that these high-melting-point long-chain alkanes precipitate as solid microcrystals at ambient and intermediate-to-high temperatures, acting as “nano-elastic fillers” within the asphalt matrix. This crystalline lattice severely restricts the relative sliding of the asphaltene and maltene molecular chains, hinders the transition of the matrix into a viscous fluid, and delivers an instantaneous Hookean elastic recovery upon unloading, thereby drastically increasing the elastic proportion (storage modulus) of the system under high-temperature and low-frequency conditions.

**Figure 6:**
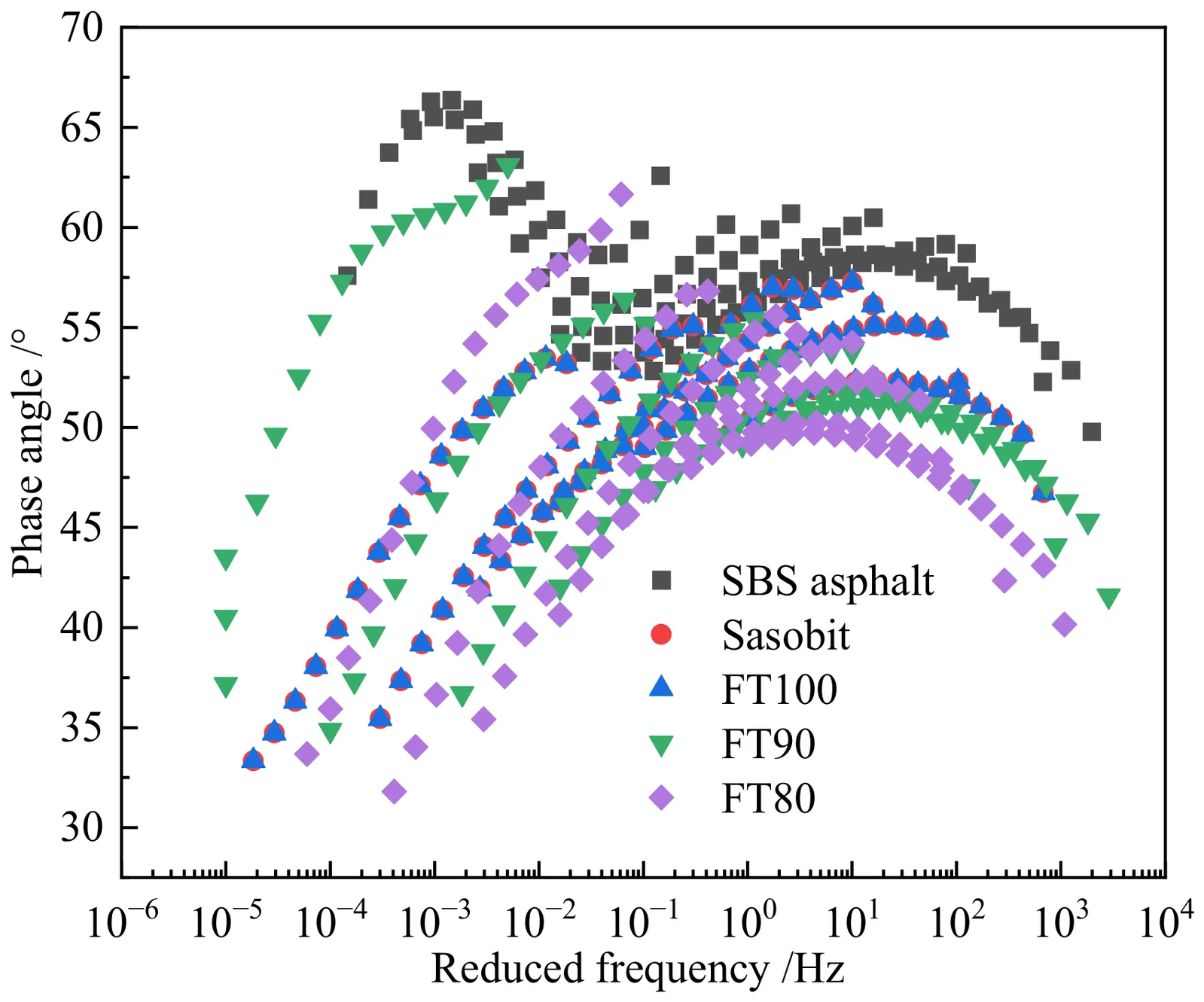
Phase Angle Master Curves of Different Modified Asphalts.

Further cross-sectional comparisons reveal that different grades of FT waxes display unique viscoelastic evolution characteristics in the low-frequency region. Although FT 80 yields the highest stiffness in the complex modulus master curve, its low-frequency phase angle (approximately 44°~55°) is markedly higher than those of Sasobit and FT 90 and FT 100. The fundamental cause of this rheological anomaly is that while FT 80 imparts high stiffness to the asphalt, the specific microcrystalline structures it forms may undergo more frequent interfacial relative sliding or micro-shear damping under low-frequency, high-shear strain conditions. This is attributed to the discontinuities at the microcrystal boundaries or variations in crystal size distribution. Globally, this microscopic slippage behavior manifests as an increased proportion of viscous dissipation. Consequently, its phase angle is not as intensely suppressed as those of Sasobit and FT 90 and FT 100, showcasing a unique viscoelastic state where high stiffness and moderate viscous dissipation coexist (Figure 6). 

### 3.5. Effect of Fischer–Tropsch Wax on the Cohesive Energy Density and Solubility Parameter of SBS-Modified Asphalt

The cohesive energy density (CED), its interaction components, and the solubility parameter (δ) of the different modified asphalts are summarized in Table 5. The incorporation of Fischer–Tropsch wax changed the CED and δ of SBS-modified asphalt to different extents, depending on the wax grade. Compared with the original SBS-modified asphalt, which showed a total CED of 2.744 × 10^8^ J/m^3^ and a solubility parameter of 16.566 (J/cm^3^)^0.5^, the addition of FT 100 increased these values to 2.762 × 10^8^ J/m^3^ and 16.619 (J/cm^3^)^0.5^, respectively. In contrast, FT 90 caused only a slight decrease in total CED and solubility parameter, whereas FT 80 reduced them more evidently to 2.726 × 10^8^ J/m^3^ and 16.510 (J/cm^3^)^0.5^. These results indicate that FT 100 enhances the molecular cohesion and compactness of the SBS-modified asphalt system, while FT 80 tends to weaken the overall intermolecular cohesion. Similar solubility-parameter differences have been reported for other small-molecule asphalt modifiers, where molecular dynamics-derived solubility parameters were likewise used to rank modifier-asphalt compatibility and to identify the chain-length or component ratio that minimizes the solubility-parameter mismatch with the base binder [38].

In terms of interaction composition, van der Waals interaction remained the dominant contributor to the cohesive energy density in all systems. For the original SBS-modified asphalt, the van der Waals contribution was 2.584 × 10^8^ J/m^3^, accounting for more than 94% of the total CED. After the addition of FT wax, the van der Waals contribution remained at a similarly high level, ranging from 2.575 × 10^8^ to 2.610 × 10^8^ J/m^3^. Among the three waxes, FT 100 produced the highest van der Waals contribution, suggesting stronger nonpolar chain packing and dispersion interactions. By contrast, FT-80 showed the lowest van der Waals contribution, implying a relatively looser molecular arrangement within the asphalt matrix.

The electrostatic interaction was much smaller than the van der Waals contribution and decreased after the addition of Fischer–Tropsch wax. The electrostatic CED decreased from 1.646 × 10^6^ J/m^3^ in the original SBS-modified asphalt to 8.936 × 10^5^, 9.187 × 10^5^, and 9.735 × 10^5^ J/m^3^ for the FT 100, FT 90, and FT 80 systems, respectively. This decrease indicates that the introduction of nonpolar wax molecules diluted or weakened the contribution of polar interactions in the asphalt system. Meanwhile, the “other” interaction term changed only slightly, remaining around 1.417 × 10^7^~1.429 × 10^7^ J/m^3^ after wax addition. Therefore, Fischer–Tropsch wax mainly affects the SBS-modified asphalt by regulating nonpolar van der Waals interactions and reducing the relative role of electrostatic interactions, rather than fundamentally changing the overall interaction type of the asphalt system.

### 3.6. Effect of Warm-Mix Additives on the Free Volume Fraction of SBS-Modified Asphalt

**Figure 7:**
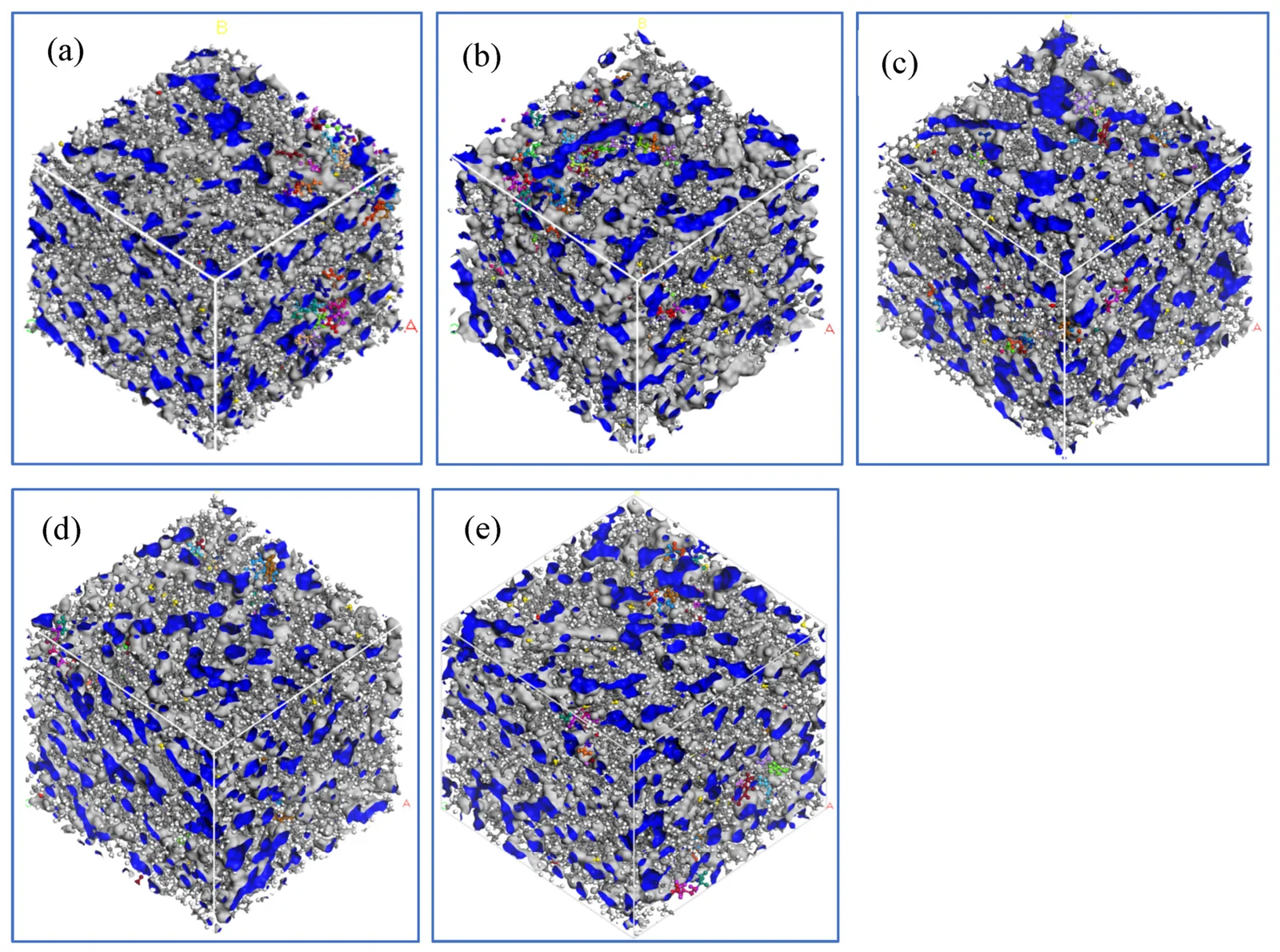
Occupied volume and free volume distribution of the asphalt–modifier composite system; The blue regions represent the free volume, whereas the gray regions represent the occupied volume: (**a**) SBS asphalt; (**b**) Sasobit; (**c**) FT 100; (**d**) FT 90; (**e**) FT 80.

The free volume fraction (FFV) results showed as Figure 7 and Table 6 indicate that warm-mix additives exerted different effects on the molecular packing structure of SBS-modified asphalt. The FFV of the original SBS-modified asphalt was 21.5507%. After the addition of Sasobit and FT 100, the FFV decreased to 21.4121% and 21.3993%, respectively, corresponding to reductions of approximately 0.64% and 0.70% compared with the original SBS-modified asphalt. This suggests that Sasobit and FT 100 reduced the free volume within the asphalt system and promoted a denser molecular arrangement. In particular, the FT 100 system exhibited the lowest FFV, indicating that it had the strongest effect on enhancing molecular packing and reducing intermolecular voids.

The FFV of the SBS-modified asphalt containing FT 90 was 21.5158%, which was only slightly lower than that of the original SBS modified asphalt. This small variation suggests that FT 90 had a relatively mild influence on the free volume structure and caused limited disturbance to the original molecular arrangement of the SBS modified asphalt. By contrast, the addition of FT 80 increased the FFV to 21.7884%, representing an increase of approximately 1.10% compared with the original SBS-modified asphalt. This indicates that FT 80 increased the intermolecular free space and produced a relatively looser molecular packing structure.

Overall, the FFV followed the order: SBS + FT 80 > SBS modified asphalt > SBS + FT90 > SBS + Sasobit > SBS + FT 100. These results suggest that Sasobit and FT 100 tend to densify the asphalt system by reducing free volume, which may contribute to improved high-temperature stability and resistance to deformation. FT 90 shows a moderate regulatory effect, whereas FT 80 increases the free volume and may enhance molecular mobility, which could be beneficial for improving fluidity and workability but may provide weaker reinforcement of the asphalt structure. This free-volume-mediated trade-off between fluidity and structural reinforcement mirrors findings from thermodynamic molecular dynamics analyses of warm-mix recycled asphalt binders, where fractional free volume was likewise used to link molecular packing changes to macroscopic softening and stiffening behavior [39].

### 3.7. Effect of Warm-Mix Additives on MSD and Diffusion Coefficient of SBS Modified Asphalt

**Figure 8:**
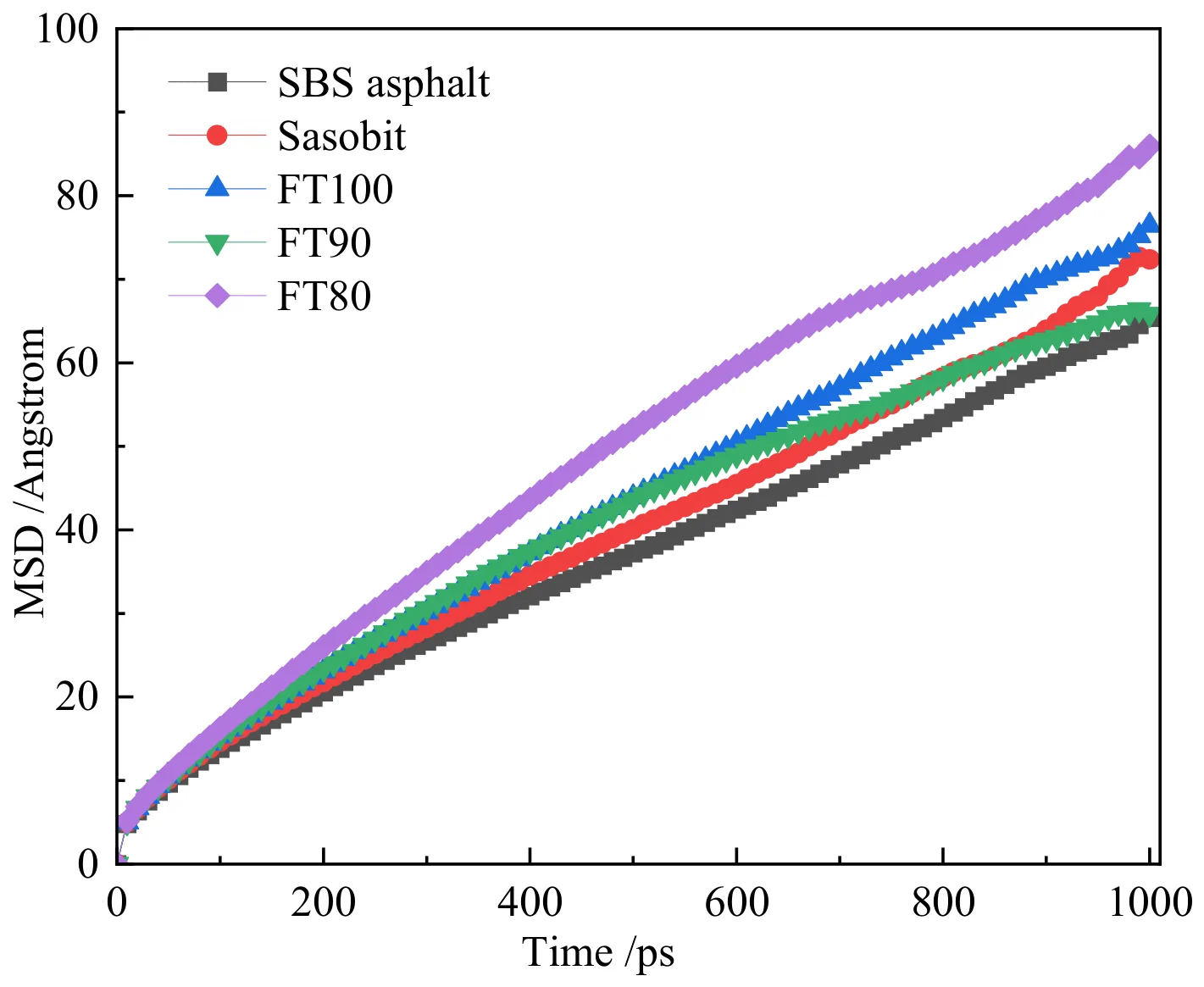
MSD Curves of Different Modified Asphalts.

The mean square displacement (MSD) results showed in Figure 8 indicate that the addition of warm-mix additives generally enhanced the molecular mobility of SBS modified asphalt. In all systems, the MSD increased continuously with simulation time, suggesting that the asphalt molecules underwent sustained diffusion during the simulation process. Compared with the original SBS-modified asphalt, the MSD values of the systems containing Sasobit, FT 100, FT 90, and FT 80 were all higher at the early stage. For example, at 100 ps, the MSD of SBS modified asphalt was 13.8318, while those of the Sasobit, FT 100, FT 90, and FT 80 systems increased to 14.6629, 15.3289, 15.2466, and 16.2233, respectively. This indicates that the warm-mix additives promoted the movement of molecular chains in the asphalt system from the beginning of the simulation.

At the end of the simulation, the differences among the systems became more evident. As showed in Table 7, at 1000 ps, the MSD of the original SBS-modified asphalt was 65.4007, whereas the MSD values of the Sasobit, FT 100, FT 90, and FT 80 systems were 72.3746, 76.4439, 65.8317, and 85.9236, respectively. Compared with the original SBS-modified asphalt, the MSD increased by approximately 10.66% for Sasobit, 16.89% for FT 100, 0.66% for FT 90, and 31.38% for FT 80. Therefore, the ability of the warm-mix additives to enhance molecular mobility followed the order: FT 80 > FT 100 > Sasobit > FT 90.

These results suggest that different warm-mix additives affect the diffusion behavior of SBS-modified asphalt through different molecular mechanisms. FT 80 showed the strongest promotion effect on MSD, indicating that it significantly increased molecular mobility and may have the greatest potential for viscosity reduction and workability improvement. FT 100 and Sasobit also enhanced molecular movement, suggesting that they can improve the fluidity of SBS modified asphalt to a certain extent. In contrast, the MSD of the FT 90 system was very close to that of the original SBS-modified asphalt at the end of the simulation, indicating that its effect on long-term molecular diffusion was relatively weak. Overall, the increase in MSD provides molecular-level evidence that warm-mix additives can improve the mobility of asphalt molecules and contribute to the warm-mix effect.

The diffusion coefficient results presented in Table 7 further demonstrate that warm-mix additives enhance the molecular mobility of SBS-modified asphalt. The diffusion coefficients were calculated from the slopes of the quasi-linear regions of the MSD curves between 200 and 800 ps using (D = k/6). The diffusion coefficient of the original SBS-modified asphalt was 0.008915 Å^2^/ps, whereas those of the Sasobit-, FT 100-, FT 90-, and FT 80-modified systems were 0.009899, 0.011213, 0.009570, and 0.012856 Å^2^/ps, respectively. Compared with the original SBS-modified asphalt, these values increased by approximately 11.04%, 25.78%, 7.35%, and 44.21%, respectively. These results indicate that all the investigated warm-mix additives promoted molecular diffusion within the SBS-modified asphalt matrix and improved molecular mobility.

Among the different additives, FT 80 exhibited the highest effective diffusion coefficient, indicating the strongest enhancement of molecular mobility within the selected fitting interval. The diffusion coefficients followed the order FT 80 > FT 100 > Sasobit > FT 90 > SBS-modified asphalt. This ranking is consistent with the relative slopes of the corresponding MSD curves. The coefficients of determination ((R^2)) ranged from 0.99125 to 0.99979, indicating good linearity of the MSD data within the selected interval of 200–800 ps. Nevertheless, because the diffusion coefficients were derived from a finite quasi-linear region rather than a strictly asymptotic long-time regime, they are interpreted here as effective diffusion coefficients.

Overall, the increases in the effective diffusion coefficients suggest that warm-mix additives can enhance molecular mobility and reduce diffusion resistance in SBS-modified asphalt, providing molecular-level evidence for their viscosity-reducing and workability-enhancing effects. Recent molecular dynamics studies of warm-mix recycled asphalt binders have reached similar diffusion-based interpretations by using mean squared displacement and diffusion coefficients to characterize the effects of warm-mix additives and rejuvenators on molecular mobility and thermodynamic behavior within the asphalt matrix [40]. Diffusion-mapping studies have likewise associated molecular diffusion behavior in warm-mix systems with improvements in mixture-level high- and low-temperature performance [41].

The rankings of the macroscopic complex shear modulus and the molecular diffusion coefficient were not fully consistent. In the low-frequency region, the stiffening effect followed the order FT 80 > FT 90 > FT 100 ≈ Sasobit, whereas the effective diffusion coefficient followed the order FT 80 > FT 100 > Sasobit > FT 90. This difference indicates that the complex shear modulus and molecular diffusivity characterize distinct aspects of the modified asphalt systems and should not be regarded as directly interchangeable indicators. The complex shear modulus reflects the macroscopic, temperature- and frequency-dependent viscoelastic response under oscillatory shear, whereas the diffusion coefficient represents molecular translational mobility within the simulated system.

## 4. Conclusions

This study investigated the effects of Sasobit and three FT waxes with different molecular chain lengths on the conventional properties, viscosity–temperature behavior, rheological performance, and molecular characteristics of SBS-modified asphalt. Based on the experimental results and molecular dynamics simulations, the following conclusions can be drawn.

The incorporation of all wax-based warm-mix additives increased the stiffness of SBS-modified asphalt. Penetration and low-temperature ductility decreased, whereas the softening point increased. These results indicate that FT waxes can improve high-temperature deformation resistance, but excessive stiffening may adversely affect low-temperature crack resistance.

All additives effectively reduced the viscosity of SBS-modified asphalt at construction temperatures, confirming their warm-mix potential. Sasobit showed the strongest viscosity reducing effect, particularly above 165 °C, where the viscosity reduction exceeded 70% compared with the control binder. FT 90 and FT 100 exhibited comparatively stable viscosity–temperature relationships, suggesting a wider and more controllable construction temperature window.

Dynamic shear rheometer results showed that the warm-mix additives increased the complex modulus and generally reduced the phase angle in the intermediate and low frequency regions. These results indicate increased binder stiffness and suggest a greater elastic contribution under the tested rheological conditions. FT 80 produced the greatest stiffness enhancement, whereas Sasobit, FT 90, and FT 100 showed more pronounced reductions in phase angle, suggesting a greater elastic contribution in the corresponding frequency regions.

Molecular dynamics simulations revealed that the influence of FT waxes depended strongly on molecular chain characteristics. FT 100 increased cohesive energy density and reduced free volume fraction, indicating a denser molecular arrangement and stronger intermolecular cohesion. In contrast, FT 80 increased free volume and produced a relatively looser molecular packing structure, which may favor molecular movement and workability but provide weaker structural compactness.

The MSD and diffusion coefficient analyses confirmed that all warm mix additives enhanced molecular mobility within the SBS modified asphalt matrix. This increased molecular mobility provides a molecular scale explanation for the viscosity reduction and improved construction workability observed experimentally.

Overall, the performance of SBS-modified warm-mix asphalt varied among the investigated FT wax additives. These comparisons are limited to the fixed dosage of 3.0 wt% and the specific test conditions employed in this study. Sasobit produced the greatest viscosity reduction, whereas FT 80 exhibited the highest low-frequency complex modulus. In the simplified MD models, FT 100 showed the highest cohesive energy density and the lowest fractional free volume. Because cohesive energy density and fractional free volume characterize intermolecular cohesion and molecular packing rather than dynamic shear stiffness, these molecular descriptors should not be interpreted as direct predictors of the G* ranking. These results demonstrate distinct performance trade-offs rather than the overall superiority of any single additive. Whether the same relative responses occur at other dosages remains to be determined. Future work should further evaluate dosage-dependent behavior, low-temperature cracking resistance, storage stability, aging behavior, and mixture-level pavement performance before practical engineering application.

## Figures and Tables

**Table 1:** Physical Properties of Base Asphalt and SBS-Modified Asphalt.

Technical Property	Unit	Base Asphalt	SBS-Modified Asphalt	Test Method
Penetration (25 °C, 100 g, 5 s)	0.1 mm	82	62.5	ASTM D5
Penetration Index	PI	−0.75	0.32	Calculated via ASTM D5&D36
Ductility (10 °C)	cm	97.5	46.0 (at 5 °C)	ASTM D113
Softening Point (Ring-and-Ball)	°C	45.5	87.5	ASTM D36
Dynamic Viscosity (60 °C)	Pa·s	165	2346	ASTM D2171
Solubility	%	99.73	99.61	ASTM D2042
Mass Change after TFOT	%	−0.45	0.03	ASTM D1754
Retained Penetration Ratio	%	59.4	64.5	ASTM D5 (After ASTM D1754)
Residual Ductility (10 °C)	cm	9.2	23.0 (at 5 °C)	ASTM D113 (After ASTM D1754)

**Table 2:** Physicochemical Properties of FT Waxes and Sasobit Additive.

Performance Indicator	FT 80 Wax	FT 90 Wax	FT 100 Wax	Sasobit Additive
Carbon Number Range	C_30_~C_60_	C_40_~C_80_	C_45_~C_100_	C_40_~C_115_
Average Molecular Weight	500~600	700~800	800~900	1000
Melting Point	80 °C	90 °C	100 °C	100 °C~110 °C

**Table 3:** Molecular Components of SBS Asphalt and Wax-Modified Asphalt Models.

Fractions	Molecular Name	Molecular Formula	Sample				
			SBS Asphalt	Sasobit	FT100	FT90	FT80
Saturates	Squalane	C_30_H_62_	17	17	17	17	17
	Hopane	C_29_H_50_	20	20	20	20	20
Aromatics	PHPN	C_35_H_44_	15	15	15	15	15
	DOCHN	C_30_H_46_	28	28	28	28	28
Resins	Quinolinohopane	C_40_H_59_N	7	7	7	7	7
	Thioisorenieratane	C_40_H_60_S	6	6	6	6	6
	Benzobisbenzothiophene	C_18_H_10_S_2_	28	28	28	28	28
	Pyridinohopane	C_36_H_57_N	8	8	8	8	8
	Trimethybenzene-oxane	C_29_H_50_O	10	10	10	10	10
Asphaltenes	Asphaltene-phenol	C_42_H_50_O	3	3	3	3	3
	Asphaltene-pyrrole	C_66_H_81_N	1	1	1	1	1
	Asphaltene-thiophene	C_51_H_62_S	1	1	1	1	1
SBS		C_108_H_124_	3	3	3	3	3
Sasobit		C_80_H_162_	0	6	0	0	0
FT100		C_50_H_102_	0	0	7	0	0
FT90		C_40_H_82_	0	0	0	9	0
FT80		C_30_H_62_	0	0	0	0	10

**Table 4:** Shift factors of different asphalt binders at various temperatures (reference temperature: 40 °C).

Asphalt	20 °C	25 °C	30 °C	40 °C	50 °C	60 °C	70 °C
SBS Asphalt	2.152	1.584	0.741	0	−0.812	−1.789	−2.864
Sasobit	2.148	1.632	0.814	0	−0.765	−1.724	−2.937
FT 80	1.832	1.258	0.645	0	−0.732	−1.585	−2.414
FT 90	2.257	1.748	0.915	0	−0.937	−2.181	−3.5
FT 100	2.148	1.632	0.814	0	−0.765	−1.724	−2.937

**Table 5:** CED and δ of Different Modified Asphalts.

Sample	CED /10^8^ J·m^−3^	van der Waals /10^8^ J·m^−3^	Electrostatic Interaction /10^6^ J·m^−3^	Other Interaction/10^7^ J·m^−3^	δ /(J·cm^−3^)^0.5^
SBS asphalt	2.744	2.584	1.646	1.439	16.566
Sasobit	2.745	2.592	1.145	1.42	16.568
FT 100	2.762	2.61	0.894	1.429	16.619
FT 90	2.739	2.588	0.919	1.422	16.550
FT 80	2.726	2.575	0.974	1.417	16.510

**Table 6:** Free Volume Characteristics of Different Modified Asphalts.

Sample	Total Occupied Volume/A^3^	Total Surface Area/A^2^	Total Volume/ A^3^	FFV /%	Relative Change Compared with SBS
SBS asphalt	90,590	39,364	115,479	21.55	/
Sasobit	97,981	42,650	124,682	21.41	−0.64%
FT 100	95,222	41,337	121,149	21.40	−0.70%
FT 90	94,305	41,068	120,162	21.52	−0.16%
FT 80	94,695	41,352	121,078	21.79	1.10%

**Table 7:** MSD and Diffusion Coefficients of Different Modified Asphalts.

Sample	MSD _t=1000_ /Angstrom	Relative Change Compared with SBS	D /Å^2^·ps^−1^	Relative Change Compared with SBS	R^2^
SBS asphalt	65.4007	—	0.008915	—	0.99962
Sasobit	72.3746	10.66%	0.009899	11.04%	0.99929
FT100	76.4439	16.89%	0.011213	25.78%	0.99979
FT90	65.8317	0.66%	0.00957	7.35%	0.99125
FT80	85.9236	31.38%	0.012856	44.21%	0.99317

## Data Availability

The original contributions presented in this study are included in the article. Further inquiries can be directed to the corresponding authors.
